# More Than Movement: Exploring Motor Simulation, Creativity, and Function in Co-developed Dance for Parkinson’s

**DOI:** 10.3389/fpsyg.2022.731264

**Published:** 2022-02-28

**Authors:** Judith Bek, Aline I. Arakaki, Fleur Derbyshire-Fox, Gayathri Ganapathy, Matthew Sullivan, Ellen Poliakoff

**Affiliations:** ^1^Division of Neuroscience and Experimental Psychology, School of Biological Sciences, University of Manchester, Manchester, United Kingdom; ^2^English National Ballet, London, United Kingdom; ^3^Equilibrium International Limited, Manchester, United Kingdom; ^4^School of Science and the Environment, Manchester Metropolitan University, Manchester, United Kingdom

**Keywords:** Parkinson’s disease, dance and movement, neurorehabilitation, motor imagery, alternative therapies

## Abstract

Dance is an enjoyable, non-therapy-focused activity that may provide a range of benefits for people with Parkinson’s. The internal simulation of movement through observation, imitation, and imagery, is intrinsic to dance and may contribute to functional improvements for people with Parkinson’s. This study explored the feasibility and potential benefits of a dance program designed by a collaborative team of dance artists, researchers, physiotherapists, and people living with Parkinson’s. The program incorporated motor simulation through observation, imitation and imagery of movement, supported by creative themes, expression, and music. A 6-week pilot trial of the program was conducted with 10 people with Parkinson’s. A focus group following the trial (*N* = 8) provided insights into the use of imagery, observation and imitation within dance, and the link between creativity and functional outcomes, as well as indicating multidimensional benefits of dance as reported in previous studies. Exploratory outcome measures also suggested potential effects on motor simulation, functional dexterity, and quality of life. The present study demonstrates the feasibility of a co-developed dance program for Parkinson’s and indicates how creative elements of dance may support functional outcomes. Future research should examine the role of motor simulation processes in dance for Parkinson’s, including the potential to develop transferable cognitive-motor skills. This study also highlights the value of collaborative partnerships in designing dance for health programs, which may optimise beneficial effects by using creative approaches to incorporate evidence-based elements, with guidance from individuals with lived experience to ensure the relevance to their goals.

## Introduction

Parkinson’s disease is the fastest growing neurological condition worldwide (Dorsey et al., 2018). Symptoms include progressive movement difficulties such as slowness, rigidity, tremor, and problems with balance and walking. There are also more subtle effects, such as difficulties with hand movements (dexterity; Foki et al., 2016), gestures (Cleary et al., 2011), and facial expressions (Bologna et al., 2013). Further to these motor impairments, Parkinson’s also causes a range of cognitive, affective, and behavioural changes (Schapira et al., 2017). Both motor and non-motor symptoms can impact significantly on activities of daily living and independence. There is currently no cure for Parkinson’s, and treatment typically involves medication to increase levels of dopamine in the brain. While medications can be effective, they do not address all symptoms and can have debilitating side-effects, necessitating the exploration of alternative, non-pharmacological approaches to enable people to live well with Parkinson’s (Li et al., 2016).

Dance involves multiple elements (including cognitive and motor skills, creativity, expression, and rhythm) that may contribute to positive effects for people with Parkinson’s (Dhami et al., 2015). Importantly, levels of physical activity (van Nimwegen et al., 2011) and motivation to exercise (Afshari et al., 2017) are reduced among people with Parkinson’s, yet high levels of adherence and motivation are found for dance (Houston and McGill, 2013; Sharp and Hewitt, 2014), reinforcing its potential as a sustainable option to help maintain health and wellbeing. Participants and instructors of dance for people with Parkinson’s have described the importance of the aesthetic experience of dance (Rocha et al., 2017; Fontanesi and DeSouza, 2021), while participants also consider addressing motor symptoms to be an important outcome (Rocha et al., 2017). The creative experience and non-therapeutic focus of dance, alongside its potential to improve movement, may result in higher levels of acceptance and motivation relative to other therapeutic activities and exercise programs for people with Parkinson’s.

Beneficial effects of various styles of dance (including Ballet, Tango, Irish, modern, and mixed styles) have been found in people with Parkinson’s, with systematic reviews and meta-analyses reporting positive effects, particularly in sensorimotor outcomes such as balance and gait (Sharp and Hewitt, 2014; dos Santos Delabary et al., 2018; Kalyani et al., 2019; Carapellotti et al., 2020). While the majority of quantitative studies have focused on physical outcomes, qualitative research also indicates a range of psychosocial benefits of dance for people with Parkinson’s, such as increased confidence and social participation, which may further contribute to its sustainability as a therapeutic activity (e.g., Westheimer, 2008; Foster et al., 2013; Houston and McGill, 2013; Westheimer et al., 2015; Bognar et al., 2017; Rocha et al., 2017; Zafar et al., 2017). However, most studies have not examined the effects of dance on everyday functional tasks and communication, or the potential to develop transferable cognitive-motor skills through dance.

There is increasing interest in understanding the mechanisms by which dance produces positive outcomes for people with Parkinson’s. The use of music within dance for people with Parkinson’s likely has a key role in supporting movement in a number of ways, including rhythmic stimulation and movement priming and cueing (Thaut and Hoemberg, 2014; Rocha et al., 2017; Ghai et al., 2018; Rose et al., 2019) as well as having emotional, motivational, and cognitive effects (Pacchetti et al., 2000; Karageorghis et al., 2020; Krotinger and Loui, 2021). It has also been hypothesised that the internal representation of movement in dance – through observation, imitation, and imagery – may contribute to some of the reported benefits for people with Parkinson’s (Bek et al., 2020). Observing and imagining movement (referred to as *action observation* and *motor imagery* in the cognitive neuroscience literature) activate frontoparietal brain areas that are involved in motor preparation and execution (Hardwick et al., 2018). These processes are intrinsic to dance, since dancers use observation and imitation to learn from and interact with others, and imagery to enhance movement quality (e.g., Blasing et al., 2012; Batson and Sentler, 2017). Dance may also influence the ability to imagine movements (Jola et al., 2011; Bar and DeSouza, 2016). Evidence from laboratory and intervention studies indicates that action observation and imagery can facilitate movement in people with Parkinson’s (Abbruzzese et al., 2015; Caligiore et al., 2017; Bek et al., 2018; Temporiti et al., 2020), with motor imagery described as a form of cognitive cueing (Keus et al., 2007). Dance may provide a more engaging and motivating context in which to develop and implement motor simulation skills than task-specific training (Bek et al., 2020), potentially offering a sustainable option for cognitive-motor rehabilitation. However, although dance programs designed for Parkinson’s frequently utilise imitation (or “mirroring”) and imagery (e.g., Hackney et al., 2007; Houston and McGill, 2013), the mechanistic role of these ostensibly aesthetic elements has received little research attention.

In summary, dance is a potentially sustainable activity within which to develop and apply cognitive-motor skills via imagery and imitation, which may contribute to functional benefits for people with Parkinson’s. To further optimise engagement and motivation, the present study involved people living with Parkinson’s in the development of a dance program, embedding motor simulation exercises within a creative framework in a way that aimed to preserve the aesthetic experience. The importance of co-developing interventions for health and physical activity is widely acknowledged (Donaldson and Finch, 2012; Buckley et al., 2018), and more specifically the value of involving people with Parkinson’s in the development process has been reported (Quinn et al., 2010; Bek et al., 2016). This interdisciplinary project drew upon knowledge from cognitive neuroscience, physiotherapy, dance for health, and lived experience of people with Parkinson’s, to explore the feasibility and potential outcomes of a co-developed dance program incorporating motor simulation processes through creative elements of dance.

## Methods

The study consisted of a co-development phase, a pilot trial, and a focus group with participants from the trial.

### The Co-development Process

The project team included researchers, dance artists and facilitators, physiotherapists and four individuals living with Parkinson’s, one of whom had an established relationship with the researchers as a patient and public involvement collaborator (MS). The researchers had previously attended and participated in dance classes for people with Parkinson’s to obtain initial insights into the experience.

The dance program was co-developed through a series of group discussions and practical sessions. The development sessions and dance classes took place at a local community arts centre, which was considered to provide a more neutral and conducive environment than a university venue or professional dance studio. Influences were drawn from English National Ballet’s (ENB) established Dance for Parkinson’s initiative and the classical Indian style Bharatanatyam. While various different types of dance have been associated with benefits for people with Parkinson’s, as noted above, Ballet and Bharatanatyam involve elements that were expected to promote motor simulation through imagery and expression. The ENB Dance for Parkinson’s model uses imagery to evoke different movement qualities, as well as communication through story-telling (Houston and McGill, 2013). Bharatanatyam is a highly expressive dance form, which also strongly features story-telling, and utilises expressive facial movements and gestures (Ponmanadiyil and Woolhouse, 2018). Recent research has suggested the potential of Bharatanatyam to provide therapeutic effects and improve coordination, concentration, and expression (Shenoy and Kumar, 2019).

**FIGURE 1:**
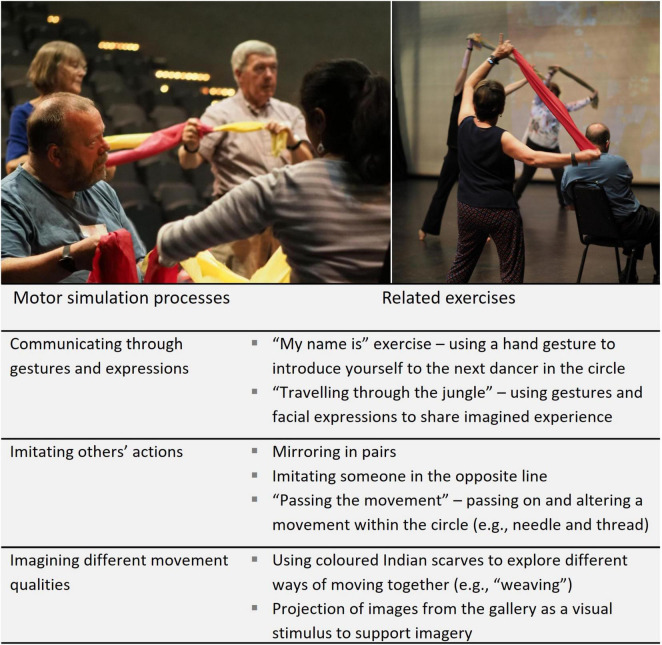
Examples of how motor simulation was promoted within the dance classes by incorporating imagery and imitation into creative exercises (photography: Chris Currie).

An important part of the development process was to identify and connect with themes to promote imagery and creativity within the dance classes. Dance is often influenced by the visual arts (e.g., Meglin et al., 2017), and to facilitate collaboration in the creative process, the development work included a visit to a gallery where team members (people with Parkinson’s, dance artists, physiotherapists, and researchers) selected and discussed artworks and exhibits as the basis for choreographic themes. A particular insight was prompted by images of spliced and obscured faces (by John Stezaker), which led to discussion of how facial expressions are affected by Parkinson’s (hypomimia), and as one individual with Parkinson’s noted “what’s in your head isn’t showing on your face.” Parkinson’s can also impair the ability to recognise emotional expressions in others, which may be related to difficulty in producing expressions (Bologna et al., 2013). Other individuals’ comments reinforced the importance of maintaining the ability to communicate non-verbally through expressions and gestures, which became a focus in the design of motor simulation exercises within the dance classes. A theme was developed around works from the gallery that linked to the local cotton industry, with complementary narratives and music evoking the environments of the cotton mill (e.g., machinery, weaving, textiles) and the Indian jungle (e.g., trees, animals, exploring the environment). The themes and music, as well as visual stimuli (images of textiles and prints from the gallery) and props including coloured scarves and handbells (see Figure 1), were incorporated to support imagery of different movement qualities.

In developing content for the classes, the dance artists emphasised the importance of preserving creative and aesthetic aspects of the experience, and the need for the research protocol to be sensitive to this. Motor simulation exercises with imitation and imagery were introduced subtly to avoid drawing attention to the research agenda (Figure 1). This was achieved through methods such as (i) projecting images from the gallery visit onto the theatre wall during classes as a stimulus for imagining different movement qualities; (ii) designing exercises to embed communicative gestures and expressions drawing upon imagery; and (iii) discreet imitation or *mirroring* by choosing someone in the opposite line to copy, or “passing on the movement” in a circle. The team members with Parkinson’s stressed the importance of balancing functional benefits with the opportunity to enjoy a creative experience that did not explicitly focus on their condition. The creativity, themes, and imagery were noted to help distinguish dance from “just sitting doing exercises.” The need for repetition and challenge to enable learning and progression was highlighted (see also Houston and McGill, 2013; Rocha et al., 2017), alongside some concerns about safety and confidence during particular movements involving balance or changes in rhythm or direction. It was also suggested that layers of complexity should be built up over time. For example, one individual noted that learning new movements initially demanded his full attention, and that appreciation of the music and using imagery would come later.

### Participants

The program was piloted with 10 individuals (7 females) aged 50–71 years with mild to moderate Parkinson’s (Hoehn and Yahr stages 1–3). Three of these participants had also been involved in the development process described above. The spouse of one participant (who did not have Parkinson’s) also joined in the classes with the agreement of the other participants, but was not included in the assessments. Two participants (1 female) were unable to attend the post-trial assessment and focus group due to unrelated health issues. The study was approved by the University of Manchester Research Ethics Committee and all participants provided written informed consent.

### Pilot Trial and Focus Group

The pilot program was delivered in 6 weekly classes of 60–75 min, in which participants were encouraged to contribute further to the choreography, and adaptations were offered for those remaining seated. Classes were followed by refreshments and social time, providing an opportunity for participants to ask questions and share feedback with the research team.

Feasibility of the dance program was assessed in terms of attendance and adverse events, as well as qualitative data from the focus group. Exploratory outcome measures were collected in the university laboratory before and after the 6-week pilot (within 7 days before the first session and after the final session). To explore whether dance participation may influence the ability to imagine movements and to simulate observed movements, which could potentially be applied outside of the dance class to aid movement in everyday life, the following measures were included: (i) a motor imagery vividness questionnaire (KVIQ; Malouin et al., 2007) which is validated for people with Parkinson’s (Randhawa et al., 2010), and (ii) a task designed to examine embodiment of observed actions, in which participants observed short videos of dance movements while their eye movements were recorded using an eye-tracker (Eyelink 1000 Plus; SR Research Ltd.), and then rated the extent to which they experienced the “feeling” of the movement on a five-point scale. To explore potential functional improvements, a self-report measure of dexterity for everyday activities (DextQ-24; Vanbellingen et al., 2016) was included, that has shown preliminary evidence of improvement following home-based motor simulation training in Parkinson’s (Bek et al., 2021a). Finally, health-related quality of life was measured using the Parkinson’s Disease Questionnaire (PDQ-39; Peto et al., 1995).

Following the trial, a focus group with participants further explored feasibility through topics such as views on different elements of the classes and perceived physical and non-physical effects. The focus group was facilitated by two members of the research team who were not involved in recruitment or collecting outcome measures (AA, EP).

The focus group was transcribed by a professional transcription service. Themes and sub-themes were identified using a combined hypothesis- and data-driven thematic analysis approach (Braun and Clarke, 2006), with the aim of exploring specific topics (experience and enjoyment of classes, motor simulation, and potential benefits) while also allowing for other aspects of participants’ experience to emerge. The transcription was coded first by one of the authors (AA); codes and initial themes were then reviewed by a second author (JB), and themes were further refined and finalised through discussions with a third author (EP).

## Results

### Feasibility and Exploratory Outcomes

The number of participants attending classes each week ranged from 6 to 9, with 7 participants attending at least 5 out of 6 classes. Non-attendance was attributed to unrelated medical appointments and other commitments. No adverse events were reported during the trial.

Statistical analysis of the exploratory pre/post outcomes was not performed because of the small sample size, but descriptive data are provided in the Supplementary Material. There was no evidence of change in self-reported motor imagery vividness (KVIQ; median −0.54%). Participants rated their sense of embodiment (“feeling”) when watching dance video clips more highly (median 9.8% change) after the dance trial, and an increase in the amplitude of saccadic eye movements (median 14.7%) suggested potential changes in how movements were observed. Change scores on self-report measures indicated potential improvements in functional dexterity (DextQ-24; median 8.9%) and quality of life (PDQ-39; median 29.8%).

### Focus Group

The following themes were generated from the focus group discussions: (1) The impact of creativity and imagery; (2) Using observation and imitation to support movement; (3) Participation in research; (4) Strength and support provided by the group; (5) Importance of the instructors and environment; and (6) Physical, emotional, and psychological effects of participation.

The themes are summarised in Table 1 and reported fully in the Supplementary Material.

**TABLE 1:** Themes generated from the post-trial focus group (participant numbers are provided after quotes where these were available from the transcript).

**Theme 1: The impact of creativity and imagery**
Participants valued being involved in a creative process that they were able to enjoy without focusing on their condition, which provided a different experience compared to typical forms of exercise.
*I like the fact it wasn’t explicit*… *I really wanted it to be like an exercise class that’s going to help to do this, it was woven in very cleverly and made you just feel like you were creating something and enjoying it, and just being immersed in it, rather than this will do you good. [F7]*
It was suggested that the creative aspects of dance could contribute to physical benefits. One individual found that imagining and using hand gestures learned during the trial had enabled them to control their movements better when communicating and performing everyday tasks:
*I thought the combination of music and the background and the story telling and all that, I loved the fact it was very creative using your imagination and yet actually physically some of the things that you did unknowingly helped me.*
*The lotus flower thing*… *it does it helps me in my job the way that I hold my hands*…*because I’ve got to present a lot of things. It stops me shaking when I do it, I don’t know why or when I unscrew bottles I think about it. [F7]*
**Theme 2: Using observation and imitation to support movement**
Participants reported a sense of embodiment through observing and imitating the instructor’s movements, such as the intricate hand gestures (mudras):
*She was so beautiful with her hands wasn’t she? I felt watching her that I was doing it like she was, I probably wasn’t, none of us probably were, but. [F3]*
The subtle use of imitation encouraged participants to explore different movements without feeling under pressure:
*I’d seen one of them doing something…you sit there thinking I can’t do that but I can have a go because nobody knows.*
One participant noticed that observing others’ movement had been helpful outside of classes while walking:
*I had one woman walking in front of me because the path was quite narrow. So she was walking ahead and I noticed that I was walking in the rhythm of her feet. [F3]*
**Theme 3: Participation in research**
It was noted that participating in research could sometimes have a negative impact by highlighting impairments, but taking part in the pilot dance program provided a sense of achievement:
*One of the things I noticed about taking part in research is when they test us for all things that they know you can’t do*…*but actually I never came away thinking I can’t…I’ve learnt a new skill.*
*I loved the fact that it was for me nothing to do with a drugs regime or an appointment regime or this is what you should be able to do. [F7]*
Some participants expressed uncertainty about the research aims and would appreciate further information on this:
*On the research side I would have liked to know more about what we’re doing.*
**Theme 4: Strength and support provided by the group.**
The importance of the group dynamics was highlighted, including a sense of support that came from being surrounded by people with similar goals and experiences:
…*thinking about being collective, it kind of gave me the strength and the courage to be able to do it…everybody’s just really putting their heart and soul into this, this is so nice. Everybody gave me encouragement. [F6]*
The supportive nature of the group also provided an encouraging and safe space for participants to express themselves:
…*it was very important that we all had Parkinson’s and people didn’t care, that was great in the sense of it didn’t put anyone off. [M3]*
**Theme 5: Importance of the instructors and environment**
Participants noted the support, sensitivity and encouragement provided by the instructors, appreciating the importance of their specialist experience and knowledge:
*I think it’s important that we do have the facilitators who actually are sensitive to our needs because if somebody comes in all gung-ho I think that would scare… [F6]*
*It was actually really clever how because they must have known beforehand that those are some of the things that people with Parkinson’s find hard. [F3]*
The venue for the dance classes (a theatre space in a community arts centre) enabled participants to immerse themselves in the creative experience without feeling self-conscious:
*It was more inspiring as well wasn’t it, you felt like you were performing rather than just in a room which was a bit cold, that was actually part of the creativity I think was to be in that space which was very helpful. [F7]*
**Theme 6: Physical, emotional, and psychological effects of participation**
This theme reflected participants’ enjoyment of the classes and the range of physical and non-physical benefits experienced. For example, some participants experienced an ease of movement while dancing:
*I was just really amazed at how I could move*… *I kept going away thinking how come I can’t walk properly but I can dance? [F6]*
Participation was also associated with an increase in motivation and confidence to try other activities:
…*we still can learn things and I think it’s easier to give up and you don’t give up. I was giving up. It’s got me going again. [F6]*
*It’s given me some confidence back*… *it’s helped me just think do you know what I can have a go at that. [F7]*

## Discussion

This study used the novel approach of co-developing and piloting a dance program for people with Parkinson’s in collaboration with dance artists and individuals living with the condition. The perspectives of the different stakeholders ensured that (i) the program was informed by scientific knowledge, (ii) evidence-based elements were incorporated sensitively to preserve the creative experience, and (iii) the needs and preferences of people with Parkinson’s were considered.

The dance program was found to be safe and enjoyable, and was well-attended apart from the impact of unrelated health issues. These findings indicate that a co-developed program is feasible. Additionally, participants reported experiencing physical and non-physical benefits of dance, and appreciated the support provided by the unique social context. The importance of the instructors’ experience and approach, and the suitability of the environment, were also emphasised. These findings are consistent with previous qualitative studies of dance for Parkinson’s (Houston and McGill, 2013; Bognar et al., 2017; Rocha et al., 2017), including the ENB Dance for Parkinson’s program (Houston and McGill, 2013).

Analysis of the focus group also found that participants were aware of using motor simulation processes in the dance classes, supported by the creative environment and elements such as music, visual stimuli, and story-telling. Action observation, imitation, and imagery were noted to influence movement both within and outside of classes, suggesting the potential transfer of motor simulation to everyday activities and communicative actions. Recent survey evidence has associated the use of imagery within dance for Parkinson’s with greater perceived benefits (Bek et al., 2021b), and preliminary findings from home-based observation and imagery training has also indicated the potential for people with Parkinson’s to develop cognitive-motor skills that can be applied more generally (Bek et al., 2021a).

As noted above, motor simulation processes used in dance share similarities with techniques used effectively in neurorehabilitation (Blasing et al., 2012; Bek et al., 2020). Moreover, the use of music, themes, and story-telling may provide a particularly conducive atmosphere to promote imagery, potentially improving adherence and effectiveness in comparison to training that explicitly focuses on motor simulation. For example, music may enhance internal action representations by activating motor control areas of the brain (Zatorre et al., 2007). The use of visual stimuli (such as the images from the gallery in the present study) to promote imagery within dance could be further explored, for example by working with a visual artist and people with Parkinson’s to select images that may evoke different movement qualities.

Similar to previous studies (Heiberger et al., 2011; McRae et al., 2018), participants valued that dance as a creative activity provided relief from thinking about their condition and focusing on their difficulties with movement. Despite some concerns during the development process about needing to concentrate initially on learning the movements before the more creative aspects of dance could be enjoyed, the focus group revealed that participants’ enjoyment of the music and story-telling may have allowed them to attend less to the physical challenges of the class. A recent small-scale study comparing immediate outcomes of dance for Parkinson’s with an exercise class of similar intensity (Fontanesi and DeSouza, 2021) found greater physiological arousal after the dance class, as well as improved motor outcomes and self-efficacy, which the authors attributed to the aesthetic components unique to dance.

The exploratory outcome measures in the present study suggested potential improvements in quality of life, consistent with previous studies of dance for Parkinson’s (see reviews, e.g., Bek et al., 2020; Carapellotti et al., 2020). The findings also indicated the potential for improving functional dexterity, which may relate to the incorporation of hand gestures in the program (see Duncan and Earhart, 2012, for additional evidence of improvements in hand movements following dance participation). Qualitative data from the focus group also revealed that participants used imagery of hand movements learned within classes to facilitate functional tasks. These findings are consistent with preliminary evidence from a recent home training study that used motor simulation to improve functional hand movements in people with Parkinson’s (Bek et al., 2021a).

Data from the exploratory measures also suggested potential changes in action observation when watching dance, whereby larger saccades might have reflected increased prediction of the movements of the observed dancers (e.g., Diaz et al., 2013), but this should be examined in further research. Although there was no evidence of change in the vividness of participants’ motor imagery, the questionnaire-based measure used in this study only tests imagery for basic isolated movements, and future studies should examine potential changes in imagery for movements that are trained within dance (e.g., Nordin and Cumming, 2006).

To increase understanding of the role of motor simulation processes within dance for Parkinson’s, a larger trial should be conducted, using quantitative and qualitative methods to explore changes in motor imagery and action observation, as well as the transfer of motor simulation techniques trained through dance to everyday tasks. As dance intrinsically involves motor simulation (Blasing et al., 2012), the effects of incorporating specific exercises based on imagery and imitation could be examined in comparison to dance classes without these exercises, to determine whether outcomes can be enhanced. Alternatively, participants could be randomly allocated to receive supplementary motor simulation training alongside dance classes to optimise the implementation of these skills.

Finally, another novel aspect of the pilot program was the successful integration of influences from Ballet and Indian dance, providing complementary elements that are highly relevant to people with Parkinson’s (e.g., postural control, hand movements, and expressive gestures), which was described by one participant as “a lovely fusion of dance styles.” While influences of Ballet are already widely used within dance programs for people with Parkinson’s, future research should further explore the use of culturally relevant styles such as Bharatanatyam, which could increase the appeal and accessibility of dance among underserved sections of the Parkinson’s community (Kelly and Leventhal, 2021).

### Conclusion

**FIGURE 2:**
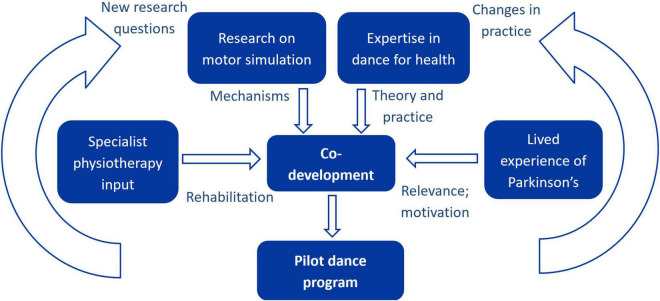
Schematic indicating contributions of the different stakeholders to the co-development process, and the feedback of knowledge from the resulting dance program into both research and practice.

This study demonstrated the feasibility and value of a co-developed dance program for people with Parkinson’s, informed by interdisciplinary expertise and lived experience. Dance for health programs can benefit from collaborative researcher-practitioner-participant partnerships (as illustrated in Figure 2), enabling evidence-based elements to be incorporated while preserving the creative and enjoyable aspects of dance. This would also empower users to have an active role in developing content and optimising the relevance to their goals. Future research should further examine the effects of motor simulation in dance for Parkinson’s, by assessing outcomes relating to cognitive-motor processes and the transfer of motor simulation skills to everyday functional movement.

## Data Availability Statement

The original contributions presented in the study are included in the article/Supplementary Material, further inquiries can be directed to the corresponding author.

## Ethics Statement

The studies involving human participants were reviewed and approved by the University of Manchester Research Ethics Committee. The patients/participants provided their written informed consent to participate in this study. Written informed consent was obtained from the individual(s) for the publication of any potentially identifiable images or data included in this article.

## Author Contributions

JB: conceptualisation, methodology, investigation, data curation, formal analysis, writing – original draft, writing, review, editing, visualisation, and project administration. AA: conceptualisation, methodology, investigation, data curation, formal analysis, writing, review, and editing. FD-F: conceptualisation, methodology, writing, review, editing, and project administration. GG: conceptualisation, methodology, investigation, writing, review, editing, and project administration. MS: conceptualisation, methodology, investigation, data curation, writing, review, and editing. EP: conceptualisation, methodology, formal analysis, writing, review, editing, visualisation, and project administration. All authors contributed to the article and approved the submitted version.

## Conflict of Interest

GG is employed by Equilibrium International Limited. FD-F is employed by English National Ballet. The remaining authors declare that the research was conducted in the absence of any commercial or financial relationships that could be construed as a potential conflict of interest.

## Publisher’s Note

All claims expressed in this article are solely those of the authors and do not necessarily represent those of their affiliated organizations, or those of the publisher, the editors and the reviewers. Any product that may be evaluated in this article, or claim that may be made by its manufacturer, is not guaranteed or endorsed by the publisher.
