# Supplementary material for: More Than Movement: Exploring Motor Simulation, Creativity, and Function in Co-developed Dance for Parkinson’s
Source: Front Psychol. 2022 Feb 28;13:731264. doi: 10.3389/fpsyg.2022.731264 (PMC8918650; doi:10.3389/fpsyg.2022.731264)
Supplement: Supplementary file 1 [file Data_Sheet_1.pdf]

Exploratory outcome measures

Exploratory outcome measures collected before (pre-trial) and after (post-trial) the 6 week pilot program. Medians and interquartile ranges are provided for pre-trial, post-trial and change (post – pre) scores.

	KVIQ-visual	KVIQ-kinesthetic	Observation of dance: embodiment rating	Observation of dance: saccade amplitude	DextQ-24	PDQ-39
Pre-trial	46.0, 16.5	44.0, 14.8	3.5, 1.0	1.8, .3	39.5, 8.8	47.0, 27.0
Post-trial	51.0, 15.8	40.0, 11.8	4.0, 1.0	2.2, .7	36.0, 11.0	35.0, 29.5
Change	-.5, 8.0	0, 9.0	.3, .6	.3, .4	-3.5, 7.0	-14.0, 11.8

KVIQ = Kinesthetic and Visual Imagery Questionnaire; DextQ-24 = Dexterity Questionnaire 24; PDQ-39 = Parkinson's Disease Questionnaire

Note: Negative change values indicate improvement on DextQ-24 and PDQ-39; positive values indicate improvement on the KVIQ.
