# Supplementary material for: More Than Movement: Exploring Motor Simulation, Creativity, and Function in Co-developed Dance for Parkinson’s
Source: Front Psychol. 2022 Feb 28;13:731264. doi: 10.3389/fpsyg.2022.731264 (PMC8918650; doi:10.3389/fpsyg.2022.731264)
Supplement: Supplementary file 2 [file Data_Sheet_2.pdf]

Themes generated from the post-trial focus group

(1) The impact of creativity and imagery

Participants valued being part of a creative process that they were able to enjoy without focusing on their condition, and commented on the impact of the themes, narrative and music, which provided a different type of experience compared to "exercise".

> *I like the fact it wasn't explicit, it...yeah, I really wanted it to be like an exercise class that's going to help to do this, it was woven in very cleverly and made you just feel like you were creating something and enjoying it, and just being immersed in it, rather than this will do you good.*
>
> *It was like a proper creative process and I think that's what I appreciated about it, rather than just Parkinson's people pretending to dance which it could have been.*
>
> *you didn't have to think about Parkinson's for five minutes*
>
> *It's like they're showing more things that help people beyond the drugs that you have to take rather than just exercise which is quite cold, this was very mentally stimulating. I love doing exercise but this was just a different side of your brain that you're using, that creative side rather than the thinking side.*
>
> *I think the stories really helped, it was brilliant to have that background.*

It was suggested that the provision of more creative movement-based activities for people with Parkinson's could increase motivation to participate:

> *...the motivation to do it. I think that's what you can easily lose and I think if more and more things were put on for people with Parkinson's, especially around movement instead of just exercise, being able to think about doing something, being creative, being artistic, being able to dance, doing music...*

The creative aspects of dance were noted to contribute to physical benefits:

> *I thought the combination of music and the background and the story telling and all that, I loved the fact it was very creative using your imagination and yet actually physically some of the things that you did unknowingly helped me.*
>
> *The weeping of the arms and the vines and all of that has had an impact.*

Participants also reported that they were able to apply imagery of movements practised within classes outside of the dance setting, to support the performance of everyday tasks:

> *Since I've been doing this I've just thought right just take your time and try and use those fine motor skills slower...That imitation and that imagery I think has been very helpful for me because I like using that side of my brain that is about that creative thing but it helps me do something functional.*

One individual had found that imagining and using hand gestures learned during the trial had enabled them to control their movements better in other settings:

> *The lotus flower thing… it does it helps me in my job the way that I hold my hands…because I've got to present a lot of things. It stops me shaking when I do it, I don't know why or when I unscrew bottles I think about it.*

I don't think right I'm going to be an elephant, but I do think there's a movement there I know I could replicate and there's movement that is helpful. So I have to put that into my conscious mind but it was really helpful for stimulating that.

(2) Using observation and imitation to support movement

Participants reported a sense of embodiment through observing and imitating the instructor's movements, such as the intricate hand gestures (mudras):

She was so beautiful with her hands wasn't she? I felt watching her that I was doing it like she was, I probably wasn't, none of us probably were but...

The subtle use of imitation encouraged participants to explore different movements without feeling under pressure:

I also like the bits where they said what...if you see an action someone else is doing that you can imitate...because I watched a few people and thought I wonder if I can do that in my head.

I found that really safe because … I'd seen one of them doing something thinking I can't do that and then you suddenly...you sit there thinking I can't do that but I can have a go because nobody knows.

The nice thing about it was we weren't competing, it didn't matter.

It was also noted that the music and the group environment could facilitate the use of imitation:

And you start copying it and you surprise yourself at what you can do with the music and the collective achievement.

One participant noticed that observing others' movement had been helpful outside of classes while walking:

I had one woman walking in front of me because...the path was quite narrow. So she was walking ahead and I noticed that I was walking in the rhythm of her feet.

(3) Participation in research

It was noted that participating in research could have a negative impact by highlighting impairments, but taking part in the pilot dance program provided a sense of achievement and was unlike other some other research projects participants had experienced:

One of the things I noticed about taking part in research is when they test us for all things that they know you can't do and then you come away thinking I can't do…but actually I never came away thinking I can't...I've learnt a new skill.

I've never enjoyed research like this, it hasn't felt like research, which is a great benefit. I've done it not because I ought to but it's interesting...it's been enjoyable as well.

I loved the fact that it was for me nothing to do with a drugs regime or an appointment regime or this is what you should be able to do.

You didn't feel as though you were being tested.

The use of wearable sensors during the classes was discussed. Initially this may have been distracting, but most participants did not notice any impact on their experience:

I think it was very distracting the first time because my thing kept looking like it was going to pop out.

The only time we were reminded of it really was when we had all to check in at the beginning.

Forgot all about them really.

Some participants expressed uncertainty about the research aims and would appreciate further information on this:

I can't see how things we were testing for before and the things we were testing for afterwards could possibly be affected by doing dance.

On the research side I would have liked to know more about what we're doing.

I'd like to know how they feel about it, what they learnt from it and what they knew about Parkinson's before and our symptoms of the condition, because everybody has a different Parkinson's and we're all moving differently.

Others were less concerned about the specific aims of the project but just wished to make a contribution to knowledge about Parkinson's:

I take a different perspective on it, I was there for the research not for the dance so it didn't trouble me at all when we went home, what we were helping, how big or small they were.

There were mixed opinions on the suggestion of providing additional information or educational materials to supplement dance training, but it was noted that resources could be made available for those who wished to access the knowledge:

[physiotherapist] came to some of our sessions and that was fascinating when she was able to link what they were planning in terms of movement with what it would do...

I think you don't think about dancing or going to dance classes. I think it's quite good to have some kind of education...

I suppose there's a delicate balance between not wanting to take away from what you do value about the class and what makes it different to attending a physio or a specific exercise.

One way around it might be the information just saying if you want to know more about this click on here or whatever, so you've got a choice then.

(4) Strength and support provided by the group

Participants commented on the importance of the group dynamics, and felt supported by being surrounded by people with similar goals and experiences:

I think it's a shared experience with like-minded people.

Absolutely, yeah. I think that's what's really important.

I felt very supported and everybody understood and supported each other so I'm very grateful.

…thinking about being collective, it kind of gave me the strength and the courage to be able to do it. But I think that was...I felt that, I felt it from everybody, I felt that I could actually...everybody's just really putting their heart and soul into this, this is so nice. Everybody gave me encouragement, yeah.

I found it incredibly reassuring that other people who were like me...I have a lot to do with Parkinson's and a lot of people are quite depressive or down, not trying to do things and it was lovely to be with a group of people who are all the same.

…positive attitude.

I feel a bit like I'm a lone voice sometimes, it was great to have other people who felt like that

I thought that from the beginning because we were... unusual in that we were all there because we wanted to do it and to help with the research. So we're not the run of the mill.

And we've learnt from each other haven't we.

The supportive nature of the group was noted as providing an encouraging and safe space for people to express themselves:

It was nice to think how uninhibited we were, nobody was frightened of making a fool.

…it was very important that we all had Parkinson's and people didn't care, that was great in the sense of it didn't put anyone off.

Views on the involvement of a spouse of one of the participants were discussed. It was agreed that it was better to have visitors join in along with the rest of the group rather than sit and observe:

He was there with his book on the first day. I loved the fact that you just threw yourself into it and you were very considerate of everybody and that just made me think we're all in this together.

But actually I think if you had have been on the sidelines just sitting watching us, that would have been quite negative, I thought it was really clever that they adapted it and you were great and went along with it and said, yeah, I'll do it as well.

…with all due respect to [participant's spouse] he wasn't that much better than us was he?

Participants valued the social aspects of the project, as well as having time after the classes to socialise:

Having something to come to that we enjoy and plus just meeting everyone that was a big thing for me.

One great thing about is meeting everybody.

..social time to say how we felt and get to know each other.

I think it was nice that we had that time at the beginning to chat and the time at the end to chat because that's part of it.

<u>(5) Importance of the instructors and environment</u>

The support, sensitivity and encouragement of the instructors were often commented on:

They were forever talking it through and they never expected us to remember a sequence.

We weren't asked to do anything that was beyond our capabilities.

Yeah, there was no pressure was there, it was...she kept saying only do as much as you feel like you can.

There weren't any put downs were there?

Get up when you're ready, get up when you want to.

I think it's important that we do have the facilitators who actually are sensitive to our needs because if somebody comes in all gung-ho I think that would scare...

It was a very level playing field wasn't it considering actually that both of them were professional dance people.

The specialist experience and knowledge of the instructors was noted to be important:

It was actually really clever how...because they must have known beforehand that those are some of the things that people with Parkinson's find hard.

What we could do and not what we can't do.

You could appreciate the work that had gone into it.

Participants enjoyed having the opportunity to contribute to the choreography:

And we could add to it if we wanted to and it was no pressure for us to add to it, it was just this gentle what you think, anybody can add a little bit.

But we did actually build on some ... that was cleverly done I think ...how we did a bit, then we repeated it more, then we went on without thinking we've got to learn this.

I think we were getting more confident to add to it as well

…kind of like an improvisation which is really good

The adaptations made by the instructors for one of the participants who remained seated were also discussed. Although it was generally agreed that seated participants were well accommodated within the group, it was suggested that this might be improved further.

They gave you the opportunity to sit down as well and do a sit down dance which was really important because not everybody's okay with getting up

you were like in the middle of everything but in a respectful way and I think if you're going to have more seated participants then that should be encouraged more. Because I think on the first week it felt a little bit like we were still finding our way with that. I don't know how it felt from your point of view but I really liked that, that we were all encouraged to be a part of it.

I thought the way that both of the group leaders led was really good actually throughout. I think the issue of the difference when you're sitting out is that it is different and I think it needs some more thinking about in terms of how to make it...it's not got to do with style of doing it or anything like that but the actual...it's literally a different thing that you end up...you're doing something that other people are not doing and that's inevitable. But I think if it was made as close as possible sometimes...some exercises that was considered rather than just a default stamp your feet then there would be more...it would feel easier to be involved from a seated position…

I like the way with you [participant] that sometimes you felt you needed to sit and sometimes you didn't and whichever you wanted they adapted it very quickly which I think was great.

The venue for the dance classes (a theatre space in a community arts centre) was found to be an important factor in enabling participants to immerse themselves in the creative experience without feeling self-conscious:

It was good to have a place that was geared to what we were doing, the sound system and the likes, draw pictures, the area that we had to work in. You didn't have to think about any of those things.

It was more inspiring as well wasn't it, you felt like you were performing rather than just in a room which was a bit cold, that was actually part of the creativity I think was to be in that space which was very helpful.

There were no distractions were there, we couldn't see out and nobody could see in.

Yeah, that's a point isn't it we didn't have anybody watching us or anything so it was really good.

(6) Physical, emotional, and psychological effects of participation.

The final theme encapsulated the range of physical and non-physical benefits that could be experienced through participating in dance.

Some participants were surprised at the ease of movement they experienced while dancing:

I was just really amazed at how I could move… I kept going away thinking how come I can't walk properly but I can dance? That's really...things like that I wish someone could say to me how can I get up and dance and then I stop and I go to walk and I can't walk.

…rhythm when we were doing all that, what did she call it, travelling, the fact that we can all style it out was amazing.

Others noted that they experienced an increase in tiredness or pain after classes, but this was not seen as a reason not to participate, since the benefits of dance were also acknowledged:

Well I think it went very well. We all got stuck in and I felt better for it at the time, me arthritic hip's complaining since, perhaps that's because we've stopped, I don't know.

I was absolutely physically exhausted by the time I got home but I was so delighted as well because I enjoyed every minute of it, it was a nice exhaustion for a change instead of just being exhausted and trying to move. Yeah, I felt really benefited by it, I miss it.

The enjoyment of the dance classes was widely acknowledged:

It was a very happy occasion.

It was a joyful experience, it made me happy. It made me smile.

Yeah, I smiled a lot, it was just really nice. We were laughing and smiling I think, oh, that's great I've been a miserable sod.

The mood did carry on.

Some participants reported that the project had also increased their motivation or confidence to try other activities:

…we still can learn things and I think it's easier to give up and you don't give up. I was giving up. It's got me going again.

It's given me some confidence back … it's helped me just think do you know what I can have a go at that. Because randomly I decided to go to walking rugby just because I thought well I can and it was because you know when we did the movement across the floor, the travelling ...I previously thought I couldn't catch a ball, throw a ball, walk, be aware of other people around me to do that well, so I'm not going because everyone else can do it. But after the dance first few weeks where we have to be aware of the space, get in between people, all of that stuff I just thought it's just a faster version of what we're doing at dance...
